# The impact of a three-dimensional interconnected continuity of care intervention on self-care capacity, negative emotional states, and quality of life in patients with ostomies

**DOI:** 10.3389/fpubh.2026.1854741

**Published:** 2026-06-17

**Authors:** Yuqing Zhang, Aihong Pan, Xufeng Wu, Peili Xu, Li Tang, Shengqin Wang

**Affiliations:** 1 Woundostomy Incontinence Clinic, Hefei First People's Hospital, Hefei, China; 2 Hospital Office, Hefei First People's Hospital, Hefei, China; 3 Nursing Department, Hospital Office, Hefei First People's Hospital, Hefei, China

**Keywords:** continuity care, negative emotions, ostomy, quality of life, self-care ability

## Abstract

**Objective:**

To evaluate the effects of a three-dimensional interconnected continuity of care intervention (hospital-community-family linkage) on self-care capacity, negative emotions, stigma, quality of life, and complication rates in patients with ostomies.

**Methods:**

A retrospective comparative study was conducted on 94 patients with intestinal or urinary ostomies treated from October 2021 to June 2024. Patients receiving routine nursing care were assigned to the control group (*n* = 47), while those receiving additional three-dimensional linked continuity care intervention were assigned to the observation group (*n* = 47). The intervention included enhanced follow-up, WeChat-based remote support, and scheduled home visits. Outcomes included compliance, self-care ability (ESCA), anxiety and depression (SAS, SDS), stigma (SIS), quality of life (Stoma-QOL), and complication rates.

**Results:**

The observation group showed significantly higher total compliance than the control group (93.62% vs. 76.60%, *p* < 0.05). After intervention, ESCA scores improved more significantly in the observation group (all *p* < 0.05). SAS and SDS scores decreased more substantially in the observation group (*p* < 0.05). SIS subscores for economic discrimination, social exclusion, social isolation, and internalized shame were significantly lower in the observation group (*p* < 0.05). Stoma-QOL scores improved significantly, while the impact of the stoma bag decreased more markedly in the observation group (*p* < 0.05). The complication rate was also significantly lower in the observation group (12.77% vs. 29.79%, *p* < 0.05).

**Conclusion:**

Compared with routine nursing care, the three-dimensional interconnected continuity of care intervention was associated with improved compliance, self-care ability, and quality of life, while reducing negative emotions, stigma, and complication risks in patients with ostomies.

## Introduction

Patients with ostomies commonly require long-term nursing care following gastrointestinal and urological surgeries (1). Due to changes in normal excretory pathways and body image, ostomy patients often experience difficulties in stoma management, skin care, and daily self-care, which can seriously affect their quality of life and increase the risk of complications such as peristomal dermatitis, stoma infection, and stoma-related inflammation (2, 3). In addition to physical dysfunction, many patients also experience significant psychological distress, including anxiety, depression, social withdrawal, and feelings of inferiority (4). These combined physical and psychological burdens make ostomy care an important challenge and focus within the healthcare system (5). Conventional nursing interventions mainly focus on in-hospital management and short-term postoperative recovery; however, long-term support and health management after discharge are often insufficient. As a result, patients may demonstrate poor compliance and inadequate self-care ability, which may negatively affect rehabilitation outcomes (6).

In recent years, continuity care models have attracted increasing attention, with the core concept focusing on extending nursing services beyond the hospital setting. Through continuous guidance and support, patients are assisted in achieving long-term self-management of their health (7). The three-dimensional linked continuity care intervention, as an innovative continuity care model, fully integrates hospital, community, and family resources, emphasizing continuity, personalization, and multidimensional support in nursing services. This model is based on patient-centered care concepts and integrated health management theories (8, 9). In this framework, hospitals provide professional medical and nursing guidance; communities serve as a bridge for care continuation and resource coordination; and families provide emotional support and daily care assistance during rehabilitation. Through close collaboration among healthcare professionals, community nurses, and family members, this multidimensional care model provides comprehensive support for ostomy patients and addresses limitations associated with traditional nursing interventions.

Previous studies (10, 11) have shown that continuity care can significantly improve treatment compliance, self-care ability, and psychological well-being in patients with chronic diseases. However, systematic research on the application of three-dimensional linked continuity care intervention in ostomy patients remains limited. Therefore, this study focused on patients with intestinal or urinary ostomies and implemented a three-dimensional linked continuity care intervention to explore its effects on patient compliance, self-care ability, negative emotions, stigma, quality of life, and complication prevention. The findings may provide clinical evidence and practical guidance for optimizing long-term nursing management and rehabilitation strategies for ostomy patients.

## Materials and methods

### Ethics

This study was conducted in accordance with the Declaration of Helsinki. The study protocol was reviewed and approved by the Ethics Committee of Hefei First People's Hospital (Approval No.: ZKWKLC-2406). Written informed consent was obtained from all participants and/or their family members before participation. All clinical data were anonymized before analysis to protect patient privacy and confidentiality.

### Study setting

This study was conducted at a tertiary general hospital in China with an established ostomy nursing service and affiliated community healthcare support system. The hospital provides specialized inpatient and outpatient ostomy care, including perioperative nursing education, discharge guidance, and follow-up management. Community healthcare services in the study region are responsible for post-discharge chronic disease management and continuity nursing support. The three-dimensional linked continuity care intervention was implemented through collaboration among hospital-based specialist nurses, community nurses, patients, and family caregivers.

### Basic data and inclusion criteria

This retrospective comparative study analyzed the clinical data of 94 patients with intestinal or urinary ostomies treated at our hospital from October 2021 to June 2024. The patients were divided into a control group (*n* = 47, receiving routine nursing interventions) and an observation group (*n* = 47, receiving three-dimensional linked continuity care intervention in addition to routine nursing care). Due to the progressive implementation of the three-dimensional linked continuity care intervention program in our department, patients admitted during the earlier clinical practice period received routine nursing care and were assigned to the control group, whereas patients admitted after implementation of the program received the three-dimensional linked continuity care intervention and were assigned to the observation group.

Inclusion Criteria: (1) Patients with a confirmed diagnosis requiring intestinal or urinary ostomy and undergoing inpatient treatment at our hospital. (2) All enrolled participants had ostomies and required long-term ostomy-related nursing care after discharge. (3) Age ≥18 years, with no restrictions on gender. (4) Ability to independently and proficiently use a smartphone. (5) Complete and authentic clinical data available for analysis. (6) Patients and their families were informed about the study and signed the relevant informed consent forms.

Exclusion Criteria: (1) Patients with severe dysfunction of vital organs such as the heart, liver, kidney, or lungs, or with malignant tumors. (2) Presence of severe immune system or hematological diseases. (3) Patients with systemic infectious diseases. (4) History of mental illness and/or cognitive impairment. (5) Allergic reactions or contraindications to the treatments or nursing interventions applied in the study. (6) Patients unable to fully cooperate with the study for any reason.

### Nursing methods

#### Control group

Routine nursing care was primarily delivered by ward nurses and specialist ostomy nurses. In this study, specialist ostomy nurses referred to nurses who had received additional standardized training in ostomy-related nursing care, including stoma assessment, peristomal skin management, stoma bag replacement, and patient education. Unless otherwise specified, the routine nursing interventions were provided to both patients and their primary family caregivers. The control group received routine nursing interventions, including: Health Education: During hospitalization, nurses provided basic health education to patients and their family members through verbal explanations and educational materials (e.g., “Stoma Care Manual”). Clean and Comfortable Environment: Nurses regularly cleaned and disinfected the ward to maintain a tidy and comfortable environment and ensured that patients had adequate rest. Psychological Support: Nurses provided emotional support and encouragement to alleviate patients’ psychological burden and improve treatment cooperation. Stoma Assessment and Care: Nurses regularly assessed and cared for the stoma, including guidance on stoma bag replacement, maintenance of peristomal skin cleanliness and dryness, and monitoring for stoma-related complications. Family Education: Family members received education regarding emotional support, companionship, common stoma-related complications, and early warning signs to improve their participation in patient care. Discharge Guidance: Before discharge, nurses provided detailed instructions regarding daily stoma care, dietary management, lifestyle adjustments, stoma bag replacement, stoma observation, and management of common stoma-related problems. Post-Discharge Follow-Up: Nurses conducted telephone follow-ups every 2 weeks to answer patients’ questions regarding home-based ostomy care, evaluate self-care ability, and monitor readmissions related to ostomy complications. Patients were also encouraged to attend outpatient follow-up visits and hospital-organized ostomy care education workshops and peer-support activities. Follow-up information in the control group was documented in routine nursing follow-up records by the responsible nurses.

#### Observation group

The observation group received three-dimensional linked continuity care interventions in addition to routine nursing care. (1) Establishment and Training of the Nursing Team: A multidisciplinary nursing team was established, consisting of one head nurse, three specialist ostomy nurses, and one community nurse. Before implementation of the intervention, all nursing staff received unified training regarding the intervention procedures, ostomy-related health education, psychological support strategies, follow-up standards, home-visit procedures, and WeChat communication protocols. The training was conducted by the head nurse through centralized lectures and case discussions to ensure consistency and standardization of the intervention process. The head nurse was responsible for intervention planning, supervision, and quality control. Specialist ostomy nurses were responsible for inpatient nursing care and discharge guidance, while community nurses conducted post-discharge follow-up and home visits. Two separate WeChat groups were established during the intervention period. One internal WeChat group was used exclusively by the nursing team for case discussion, follow-up coordination, and intervention management. Another WeChat group included nurses, patients, and primary family caregivers and was used for health education, online consultation, psychological support, and continuity care communication. (2) Standardized Nursing Interventions: ① Enhanced Follow-Up: Community nurses conducted telephone follow-ups at least twice weekly during the 3-month intervention period. Follow-up content included assessment of stoma condition, peristomal skin status, dietary management, medication adherence, rehabilitation exercises, psychological status, and self-care performance. Standardized follow-up records were maintained for all patients, and complex problems were discussed within the nursing team to optimize individualized nursing plans. All post-discharge assessments and intervention records in the observation group were documented using standardized continuity care record forms maintained by the nursing team. ② WeChat-Based Education and Support: Nurses provided online support every Wednesday and Saturday from 4:00 p.m. to 8:00 p.m. through the WeChat platform. Educational content included stoma bag replacement procedures, peristomal skin care, prevention and early identification of complications, dietary guidance, emotional management, rehabilitation exercises, and daily lifestyle management. Educational videos, illustrated manuals, and reminder messages were regularly distributed through the WeChat group. Patients and family caregivers could consult nurses regarding home-based ostomy care and receive psychological support in real time. ③ Home Visits: Home visits were conducted at 1 week, 4 weeks, 8 weeks, and 12 weeks after discharge. During each visit, nurses used a standardized home-visit checklist to evaluate stoma appearance, peristomal skin condition, stoma bag management, medication compliance, nutritional status, rehabilitation exercise performance, psychological condition, and self-care ability. Based on the assessment results, individualized nursing guidance and intervention adjustments were provided. (3) Role of Family Caregivers: Family caregivers were encouraged to actively participate in patient care throughout the intervention period. Nurses educated family members regarding emotional support, assistance with stoma care, dietary supervision, complication monitoring, and reinforcement of rehabilitation exercises and follow-up adherence. (4) Intervention Fidelity Monitoring: To ensure intervention consistency, the head nurse conducted regular supervision of follow-up records, WeChat communication logs, and home-visit documentation. All patients in the observation group received the same intervention duration, follow-up frequency, WeChat education schedule, and home-visit protocol during the 3-month intervention period. Both groups received nursing interventions for 3 months, and outcome measures were evaluated after completion of the intervention period.

### Observation indicators

1. Compliance: Compliance was evaluated 1 month after intervention by two trained nurses who were not directly involved in outcome data analysis. The assessment was conducted based on follow-up records, nursing documentation, and patient self-reports collected during telephone follow-up and home visits. Compliance evaluation included four aspects: medication adherence, dietary management, regular daily routines, and follow-up attendance. Medication adherence referred to taking prescribed medications according to medical instructions. Dietary management referred to adherence to recommended dietary guidance for ostomy patients, including appropriate fluid intake, avoidance of irritating foods, and maintenance of balanced nutrition. Regular daily routines referred to maintaining relatively stable sleep patterns, rest schedules, and daily living habits. Follow-up attendance referred to active participation in scheduled follow-up visits and nursing assessments. Patients meeting all four criteria were classified as having full compliance; those meeting three criteria were classified as having partial compliance; and those meeting two or fewer criteria were classified as having poor compliance. Total compliance rate = (Full compliance + Partial compliance cases) / Total cases × 100%.
2. Self-Care Ability: Self-care ability was assessed using the Exercise of Self-Care Agency Scale (ESCA) developed by Kearney and Fleischer (12). All questionnaires were administered in Chinese by trained nurses before and after intervention. The ESCA includes four dimensions: self-care skills, health knowledge, self-responsibility, and self-concept, with a total of 43 items scored on a 5-point scale. Scores range from 0 to 172, with higher scores indicating better self-care ability.
3. Negative Emotions: Anxiety and depression were assessed using the Chinese versions of the Self-Rating Anxiety Scale (SAS) and Self-Rating Depression Scale (SDS), which have been widely used and validated in Chinese populations (13, 14). All questionnaires were completed by patients independently under nurse guidance. Both scales have a maximum score of 100, with cutoff values of 50 for SAS and 53 for SDS. Higher scores indicate more severe negative emotions.
4. Stigma: Stigma was assessed using the Chinese version of the Social Impact Scale (SIS), which has demonstrated good reliability and validity in Chinese clinical populations (15). The scale covers four dimensions: economic discrimination, social rejection, social isolation, and internalized shame. The scale consists of 24 items scored on a 4-point scale, with a total score range of 24–96. Higher scores indicate greater stigma.
5. Quality of Life: Quality of life was assessed using the Chinese version of the Stoma Quality of Life Scale (Stoma-QOL), which has been translated and validated for use in Chinese ostomy patients (16). The questionnaires were administered in Chinese before and after intervention. The scale includes four dimensions: social interaction, family relationships, physical and mental health, and the impact of stoma bags, with 20 items scored on a 4-point scale. Total scores range from 20 to 80.
6. Complications: All stoma-related complications occurring during the 3-month intervention period were recorded by the hospital nursing team and follow-up staff. The recorded complications included stoma swelling, retraction, prolapse, bleeding, peristomal dermatitis, and urinary tract infections. Both mild and severe complications were included in the analysis. Mild complications referred to complications that could be managed through routine nursing care or outpatient treatment, whereas severe complications referred to complications requiring hospitalization, invasive treatment, or specialized medical intervention.

### Statistical analysis

GraphPad Prism 8 was used for charting, and SPSS 22.0 was used for statistical analysis. Categorical data: Expressed as percentages (%), analyzed with χ^2^ tests. Continuous data: Expressed as (x ± s). Between-group comparisons were conducted using independent sample t-tests, while paired t-tests were used for within-group comparisons before and after intervention. Given the retrospective comparative design and the exploratory nature of this study, no adjustment for multiple comparisons was performed. A *p*-value of <0.05 was considered statistically significant.

## Results

### Comparison of basic data

There were no significant differences between the two groups in terms of gender, age, etiology, disease duration, underlying diseases, or educational level (*p* > 0.05), indicating comparability. See Table 1.

**Table 1:** Comparison of clinical data (*x* ± s, n [%]).

Characteristic	Control (*n* = 47)	Observation (*n* = 47)	*t/x^2^*	*p*
Gender	-	-	0.389	0.532
Male	28 (59.57)	25 (53.19)	-	-
Female	19 (40.43)	22 (46.81)	-	-
Age (years)	60.47 ± 8.35	61.12 ± 7.89	0.387	0.699
Type of ostomy	-	-	0.518	0.471
Intestinal ostomy	34 (72.34)	37 (78.72)	-	-
Urinary ostomy	13 (27.66)	10 (21.28)	-	-
Nature of ostomy	-	-	0.721	0.396
Temporary ostomy	29 (61.70)	32 (68.09)	-	-
Permanent ostomy	18 (38.30)	15 (31.91)	-	-
Duration since ostomy creation	-	-	0.446	0.504
≤3 months	21 (44.68)	24 (51.06)	-	-
>3 months	26 (55.32)	23 (48.94)	-	-
Primary diagnosis	-	-	0.637	0.727
Colorectal cancer	25 (53.19)	27 (57.45)	-	-
Bladder cancer	10 (21.28)	8 (17.02)	-	-
Intestinal obstruction/others	12 (25.53)	12 (25.53)	-	-
Underlying diseases	-	-	-	-
Hypertension	18 (38.30)	20 (42.55)	0.176	0.674
Diabetes	14 (29.79)	17 (36.17)	0.433	0.510
Heart disease	8 (17.02)	11 (23.40)	0.593	0.441
Chronic kidney disease	5 (10.64)	6 (12.76)	0.103	0.748
Previous ostomy education	-	-	0.192	0.661
Yes	16 (34.04)	18 (38.30)	-	-
No	31 (65.96)	29 (61.70)	-	-
Educational level	-	-	0.274	0.600
High school or below	37 (78.72)	39 (82.98)	-	-
College or above	10 (21.28)	8 (17.02)	-	-

### Comparison of compliance

**Figure 1:**
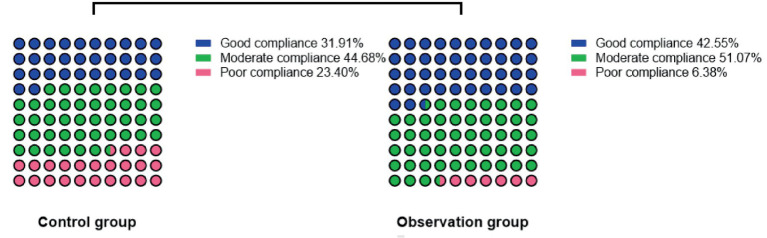
Comparison of compliance [*n*(%)]. Comparison between groups, *p < 0.05.

In the control group, 15 patients had good compliance, 21 had moderate compliance, and 11 had poor compliance. In the observation group, 20 patients had good compliance, 24 had moderate compliance, and 3 had poor compliance. The total compliance rate in the observation group (93.62%) was higher than that in the control group (76.60%; *p* < 0.05), as shown in Figure 1.

### Comparison of self-care ability

In the control group, the before and after intervention scores for self-care skills were (21.52 ± 0.63, 32. 15 ± 0.84), health knowledge scores were (31.67 ± 0.78, 36. 15 ± 0.69), self-responsibility scores were (17.49 ± 2.93, 19.67 ± 3.02), and self-concept scores were (21.65 ± 0.67, 23. 14 ± 0.83). In the observation group, the pre- and post-intervention scores for self-care skills were (21.43 ± 0.75, 36.97 ± 0.78), health knowledge scores were (31.53 ± 0.86, 42.96 ± 0.77), self-responsibility scores were (17.76 ± 3.08, 22. 15 ± 1.74), and self-concept scores were (21.58 ± 0.72, 27.97 ± 0.74).

**Figure 2:**
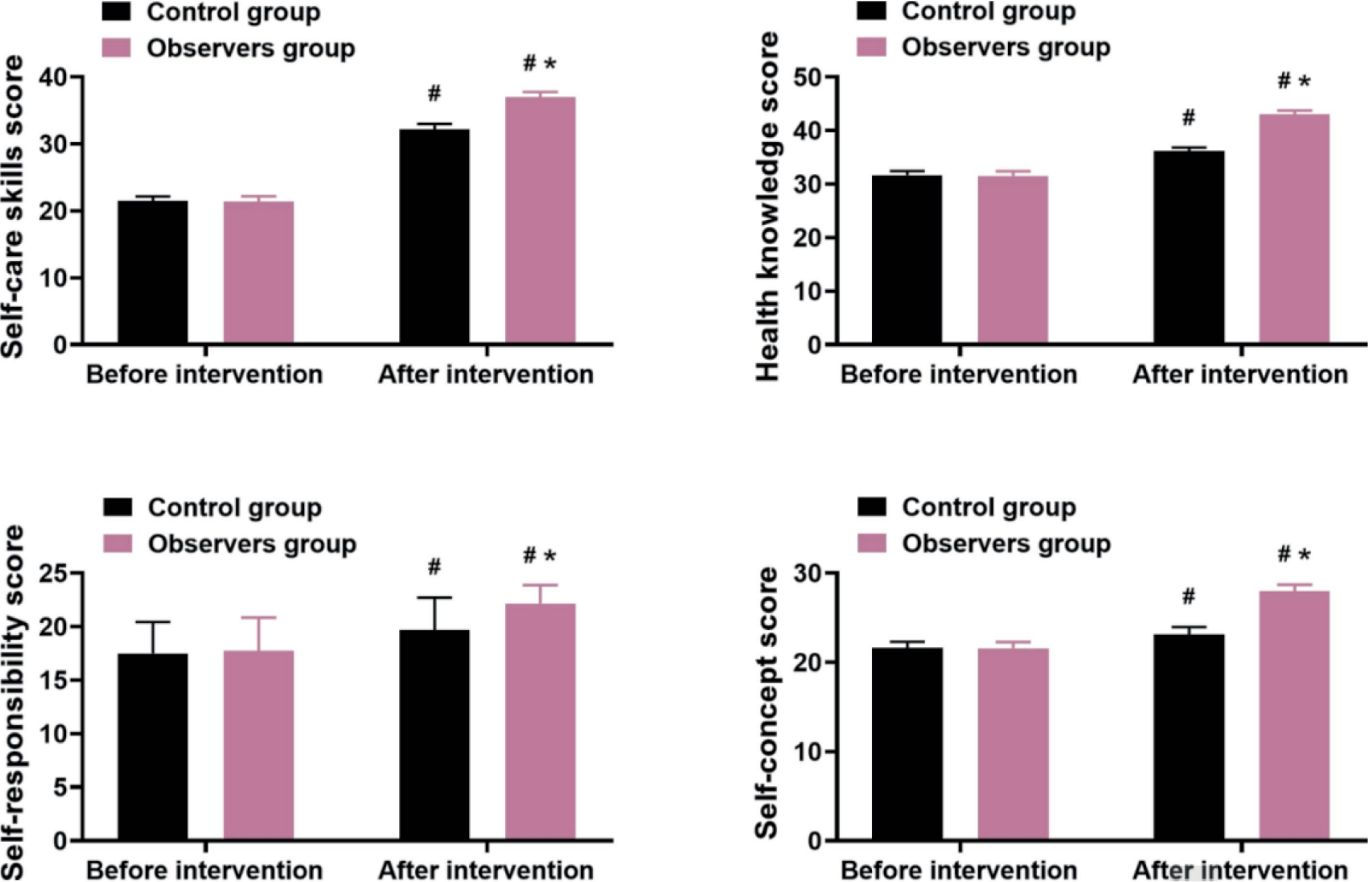
Comparison of self-care ability (*x* ± *s*, points). Comparison with before intervention, ^*^p < 0.05; Comparison between groups, #*p* < 0.05.

After intervention, the self-care skills, health knowledge, self-responsibility, and self-concept scores in both groups increased, with the observation group showing a greater improvement (*p* < 0.05), as shown in Figure 2.

### Comparison of negative emotional status

**Figure 3:**
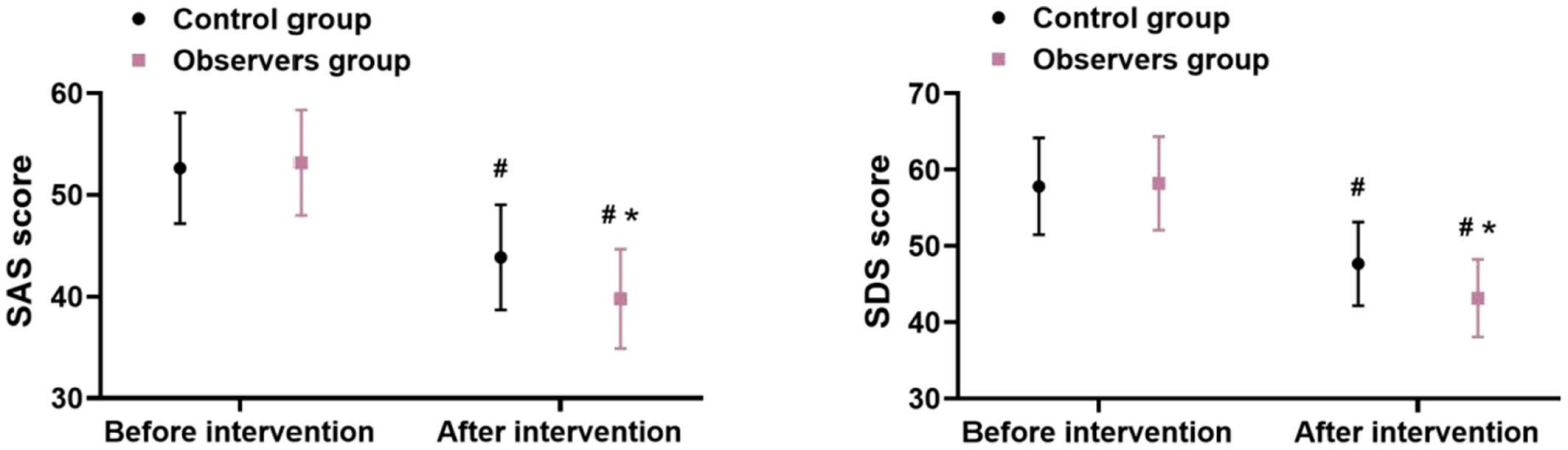
Comparison of negative emotional status (*x* ± *s*, points). Comparison with before intervention, *p < 0.05; comparison between groups, #*p* < 0.05.

In the control group, the before and after intervention SAS scores were (52.63 ± 5.45, 43.86 ± 5. 17), and SDS scores were (57.82 ± 6.35, 47.67 ± 5.49). In the observation group, the pre- and post-intervention SAS scores were (53. 17 ± 5.19, 39.78 ± 4.89), and SDS scores were (58.22 ± 6.14, 43. 16 ± 5.08). After intervention, both groups showed a decrease in SAS and SDS scores, with the observation group showing a greater reduction (p < 0.05), as shown in Figure 3.

### Comparison of stigma

After intervention, both groups showed a decrease in economic discrimination, social exclusion, social isolation, and internal shame scores, with the observation group showing a greater improvement (*p* < 0.05), as shown in Table 2.

**Table 2:** Comparison of stigma (*x* ± s, points).

Item/Time point	Control (*n* = 47)	Observation (*n* = 47)	*t*	*p*
Economic discrimination score	-	-	-	-
Before intervention	7.92 ± 1.75	7.64 ± 1.71	0.784	0.434
After intervention	6.93 ± 1.54^#^	6.05 ± 1.83^#^	2.522	0.013
Social exclusion score	-	-	-	-
Before intervention	24.67 ± 2.91	24.56 ± 2.53	0.195	0.845
After intervention	22.48 ± 3.52^#^	18.45 ± 2.87^#^	6.083	<0.001
Social isolation score	-	-	-	-
Before intervention	19.27 ± 1.96	19.56 ± 2.11	0.690	0.491
After intervention	17.58 ± 2.11^#^	14.37 ± 1.95^#^	7.659	<0.001
Internal shame score	-	-	-	-
Before intervention	15.26 ± 1.67	15.09 ± 2.02	0.444	0.657
After intervention	14.03 ± 1.85^#^	10.64 ± 1.48^#^	9.809	<0.001

Comparison with before intervention in the same group, #*p* < 0.05.

### Comparison of quality of life

After intervention, both groups showed an increase in social interaction, relationship with family, and physical and mental health scores, and a decrease in the impact of the stoma bag. The observation group showed a greater improvement (*p* < 0.05), as shown in Table 3.

**Table 3:** Comparison of quality of life (*x* ± s, points).

Item/Time point	Control (*n* = 47)	Observation (*n* = 47)	*t*	*p*
Social interaction score	-	-	-	-
Before intervention	13.47 ± 1.05	13.72 ± 1.01	1.176	0.242
After intervention	15.94 ± 1.68^#^	17.21 ± 1.75^#^	3.589	<0.001
Relationship with family score	-	-	-	-
Before intervention	12. 17 ± 1.64	12.40 ± 1.52	0.705	0.482
After intervention	14.63 ± 1.95^#^	17.39 ± 1.31^#^	8.054	<0.001
Physical and mental health score	-	-	-	-
Before intervention	5.42 ± 1.11	5.61 ± 0.96	0.887	0.377
After intervention	6.87 ± 1.29^#^	8.74 ± 1.05^#^	7.707	<0.001
Impact of stoma bag	-	-	-	-
Before intervention	11.72 ± 1.78	11.59 ± 1.67	0.365	0.715
After intervention	9.87 ± 2.16^#^	8.34 ± 1.65^#^	3.859	<0.001

Comparison with before intervention in the same group, #*p* < 0.05.

### Comparison of complications

The incidence of complications in the observation group (12.77%) was lower than that in the control group (29.79%; *p* < 0.05), as shown in Table 4.

**Table 4:** Comparison of complications [n(%)].

Complication	Control (*n* = 47)	Observation (*n* = 47)	*2 x*	*p*
Stoma swelling	3 (6.38)	1 (2. 13)	-	-
Stoma retraction	2 (4.26)	1 (2. 13)	-	-
Stoma prolapse	2 (4.26)	0 (0.00)	-	-
Stoma bleeding	3 (6.38)	2 (4.26)	-	-
Peristomal dermatitis	2 (4.26)	1 (2. 13)	-	-
Urinary tract infection	2 (4.26)	1 (2. 13)	-	-
Total incidence	14 (29.79)	6 (12.77)	4.064	0.043

## Discussion

Patients with wound stoma incontinence often face multiple challenges, including physiological, psychological, and social difficulties, due to changes in body structure and the increased complexity of nursing care (17). These patients not only need long-term management of stoma-related nursing issues but also have to cope with problems such as stoma-related stigmatization, negative emotions, and a decline in quality of life (18). Previous studies (19, 20) have shown that conventional nursing models often fail to fully address the nursing gaps encountered by patients during the later stages of hospitalization and after discharge, which impedes patient compliance, self-care ability, and physical and psychological recovery. In this context, continuity of care models has gradually gained attention, emphasizing continuous care from the hospital to the community and then to the home, aiming to achieve comprehensive health management and improvement for patients (21). The three-way linkage continuity of care intervention is an innovative nursing model, which focuses on building a multi-layered care support system through close collaboration between hospitals, communities, and families. This model aims to address the fundamental issue of care fragmentation found in traditional nursing models by providing comprehensive and efficient care services through personalized guidance, continuous psychological interventions, and active family participation.

Compliance is a key factor affecting nursing outcomes and the recovery process for patients (22). Wound stoma incontinence patients often suffer from insufficient compliance due to long-term nursing pressure, operational complexity, and psychological stress. The results of this study show that the nursing compliance of the observation group was significantly higher than that of the control group (*p* < 0.05).

This result is consistent with previous related studies (23, 24), suggesting that the three-dimensional linked continuity care intervention was associated with improved patient compliance. The reason for this may lie in the integrated role of in-hospital education, community follow-up, and family support, which not only enhances the patients’ understanding of nursing plans but also improves compliance through continuous supervision from the community and family. For example, regular follow-up and health education by community nurses can help patients correct improper nursing behaviors in a timely manner; the involvement of family members can further enhance the patients’ motivation and confidence in care. Regarding self-care ability, the patients in the observation group showed significant improvement in self-care ability after the intervention, with notable advantages in dimensions such as self-care skills, health knowledge, self-responsibility, and self-concept (*p* < 0.05). The reason for this improvement is that the three-dimensional linked continuity care intervention is patient-centered, offering personalized health education and skills training, enabling patients to independently perform stoma care. This intervention model not only emphasizes nursing skills training but also focuses particularly on improving patients’ health knowledge. Furthermore, through psychological support and behavioral guidance, patients gradually build a positive sense of self-responsibility and a positive self-concept, forming good self-management habits. This comprehensive intervention strategy enhances the patients’ self-care ability from multiple dimensions and lays a solid foundation for their long-term recovery.

Anxiety and depression are common negative emotional issues in patients with ostomy patients (25). This study found that the SAS and SDS scores of patients in the observation group after intervention were significantly lower than those in the control group (*p* < 0.05), suggesting that the three-dimensional linked continuity care intervention was associated with reduced negative emotions in patients. This can be attributed to the multi-level psychological support system. Firstly, psychological assessment and counseling during the hospital phase help patients initially accept the reality of the stoma and reduce anxiety. Secondly, long-term follow-up by community nurses provides continuous psychological counseling and emotional support for patients. Finally, family members, through their involvement in the nursing process, offer care and encouragement, thus reducing the patients’ sense of loneliness and inferiority (26). Additionally, stoma patients often face social exclusion, isolation, and economic discrimination due to changes in body image and reduced quality of life (27). In this study, the scores for economic discrimination, social exclusion, and internal shame in the observation group were significantly reduced compared to the control group (*p* < 0.05), suggesting that the three-dimensional linked continuity care intervention may be associated with improvements in stigma-related outcomes and social adaptation. This result may be closely related to the emphasis on building a social support network during the nursing intervention process. Through the integration of community resources, patients can receive more social care and assistance; through family support, patients’ self-esteem and social participation are enhanced. Regarding quality of life, this study found that the patients in the observation group had significantly higher quality of life scores (including social interaction, intimate relationships, physical and mental conditions) after the intervention, and the impact score of the stoma bag was significantly lower (*p* < 0.05). Improving quality of life is an important goal of nursing interventions, and the quality of life of patients with ostomy patients is influenced by multiple factors, including physical dysfunction, psychological burden, and social adaptation (28). The three-dimensional linked continuity care intervention may contribute to improvements in patients’ overall quality of life through comprehensive measures. For example, health education and skills training improve the patients’ physical and mental conditions; psychological support alleviates negative emotions; and collaboration from family and society enhances patients’ social interaction abilities. Finally, regarding complications, this study shows that the incidence of complications in the observation group was significantly lower than that in the control group (*p* < 0.05). The reduction in complications is likely related to the strengthened professional guidance and regular monitoring in the three-dimensional linked continuity care intervention. For instance, regular follow-up by community nurses can detect and address skin issues around the stoma early, and active participation by family members helps patients use care products correctly and prevent infections. This systematic nursing model may help reduce nursing gaps and decrease the risk of complications.

The findings of this study suggest that the three-dimensional linked continuity care intervention may have favorable applicability and potential clinical benefits in patients with ostomies. This model should be further promoted and its application in other chronic disease care should be explored. In addition, it is recommended to strengthen personalized nursing strategies in nursing practice, particularly in psychological intervention and social support, and develop targeted programs based on the specific needs of different patients to maximize nursing outcomes. It should be noted that this study still has several limitations. First, this was a single-center retrospective comparative study with a relatively small sample size, which may limit the external validity and generalizability of the findings. Second, because the study lacked randomization, potential selection bias and residual confounding factors could not be completely excluded, and causal relationships between the intervention and outcomes should therefore be interpreted with caution. In addition, several outcome indicators, including self-care ability, stigma, anxiety, depression, and quality of life, were assessed using self-reported scales, which may introduce subjective reporting bias. The follow-up duration of this study was relatively short, and the long-term sustainability of the intervention effects requires further investigation. Moreover, due to the nature of the nursing intervention, blinding of patients and nursing staff was not feasible, which may have introduced potential assessment bias. In addition, because multiple outcome indicators were compared using t-tests, the possibility of type I error could not be completely avoided. Future studies should further expand the sample size and adopt multicenter prospective randomized designs with longer follow-up periods. Repeated-measures analysis or ANCOVA adjusted for baseline values should also be considered, and effect sizes and confidence intervals should be reported to further improve statistical reliability and strengthen the evidence supporting the clinical application of this intervention model. In addition, the three-dimensional linked continuity care intervention requires substantial human resource involvement, including specialist ostomy nurses, community nurses, continuous follow-up, home visits, and WeChat-based support. Such intensive intervention models may increase nursing workload and healthcare resource utilization. Although the present study demonstrated favorable improvements in patient-related outcomes, further studies evaluating cost-effectiveness, staffing efficiency, and long-term sustainability are still needed before large-scale implementation can be recommended. Future research should also explore simplified or resource-optimized continuity care strategies to improve feasibility in healthcare settings with limited nursing resources.

## Data Availability

The original contributions presented in the study are included in the article/supplementary material, further inquiries can be directed to the corresponding author.
